# Long-Term Cognitive and Functional Outcomes of Anesthesia and Surgery in Patients with Dementia

**DOI:** 10.3390/healthcare14172821

**Published:** 2026-09-02

**Authors:** Antea Krsek, Lana Sabati, Ivana Krajina, Zofia Nikola Lewandowska, Vlatka Sotosek, Lara Baticic

**Affiliations:** 1 Faculty of Medicine, University of Rijeka, 51000 Rijeka, Croatia; tea.krsek@gmail.com (A.K.); lana.sabati@uniri.hr (L.S.); ikrajina@uniri.hr (I.K.); 2 Faculty of Medicine, Medical University of Lodz, 90-419 Łódź, Poland; zofia.lewandowska@stud.umed.lodz.pl; 3 Department of Clinical Medical Sciences I, Faculty of Health Studies, University of Rijeka, 51000 Rijeka, Croatia; 4 Department of Anesthesiology, Reanimatology and Intensive Care Medicine, Faculty of Medicine, University of Rijeka, 51000 Rijeka, Croatia; 5 Department of Medical Chemistry, Biochemistry and Clinical Chemistry, Faculty of Medicine, University of Rijeka, 51000 Rijeka, Croatia

**Keywords:** anesthesia, cognitive decline, cognitive reserve, dementia, functional outcomes, neuroinflammation, perioperative neurocognitive disorders, postoperative delirium, surgery

## Abstract

Dementia is one of the leading causes of disability and dependence among older adults and is increasingly encountered in patients undergoing surgery. As populations age and surgical interventions become more common, understanding the perioperative care of patients with pre-existing dementia has become an important clinical priority. Dementia is associated with reduced cognitive reserve, chronic neuroinflammation, synaptic dysfunction, blood–brain barrier impairment, neurotransmitter abnormalities, and cerebrovascular dysregulation, all of which may increase vulnerability to perioperative neurological injury. Surgical trauma and anaesthesia trigger systemic inflammatory, metabolic, and haemodynamic responses that may disrupt cerebral homeostasis and contribute to perioperative neurocognitive disorders, including postoperative delirium and delayed neurocognitive recovery. Postoperative delirium is consistently associated with accelerated cognitive and functional decline and may represent an important link between perioperative stress and adverse long-term outcomes. However, despite strong biological plausibility, current clinical evidence does not establish a direct causal relationship between anaesthesia, surgery, and accelerated dementia progression. Interpretation of the available literature is limited by methodological heterogeneity, residual confounding, and the frequent exclusion of patients with established cognitive impairment. This narrative review summarizes the neurobiological mechanisms underlying increased perioperative vulnerability in dementia, critically examines current evidence regarding the effects of anaesthesia and surgery on long-term cognitive and functional outcomes, and discusses practical strategies to reduce perioperative risk. A better understanding of these complex interactions is essential to optimize perioperative care and preserve cognitive function, functional independence, and quality of life in this highly vulnerable patient population.

## 1. Introduction

Dementia is one of the most important public health challenges of the 21st century. Although age-standardized incidence rates have stabilized or declined in some high-income countries, the overall number of people living with dementia continues to rise because of population aging. As life expectancy increases, dementia is becoming increasingly common among older adults requiring surgical care, making its perioperative management an important clinical priority [1,2,3]. Population aging has concurrently led to an increased prevalence of older patients requiring surgery. Dementia is now one of the most encountered comorbidities in the modern perioperative period [4,5]. Current estimates indicate that approximately one out of every four patients aged 65 years and older undergoing major surgical procedures has some level of cognitive impairment, with a significant proportion diagnosed with dementia. Cognitive impairment encompasses a spectrum ranging from mild cognitive impairment, characterized by objective cognitive decline without loss of functional independence, to dementia, which is defined by cognitive impairment accompanied by functional decline sufficient to meet established diagnostic criteria. As life expectancy increases further, anaesthesiologists and surgeons are increasingly faced with the challenge of managing patients with dementia and other neurodegenerative conditions throughout the perioperative period [6,7].

In parallel with this trend, patients with dementia are increasingly undergoing anaesthesia and surgical procedures. Urgent conditions such as hip fractures, abdominal emergencies, and cardiovascular disease require urgent surgical intervention in this patient population. Additionally, with improvements in surgical and perioperative care, patients with early and moderate dementia are now undergoing elective surgical procedures. Accordingly, anaesthesia is no longer a rare event but an increasingly routine aspect of care in patients with dementia [8,9].

Despite this epidemiological fact, patients with pre-existing dementia are still underrepresented in perioperative studies. Many clinical studies of perioperative neurocognitive disorders have excluded patients with pre-existing cognitive impairment, thereby greatly limiting the generalizability of the results to this clinically high-risk population. Therefore, the long-term cognitive and functional consequences of anaesthesia and surgery in patients with pre-existing dementia are still not well characterized [10,11,12].

The focus of attention on perioperative neurocognitive disorders in the context of pre-existing dementia is well justified from the clinical and biological points of view. Dementia is associated with reduced cognitive reserve, chronic neuroinflammation, synaptic and neuronal network dysfunction, and abnormal cerebrovascular regulation [13,14]. These pathophysiological changes may increase susceptibility to perioperative neurocognitive disorders and could potentially contribute to worse postoperative neurological outcomes. In addition, the potential association between anaesthesia, surgery, and long-term cognitive or functional decline in patients with dementia remains incompletely understood [15,16].

These neurocognitive changes occur on a continuum. While early postoperative cognitive disturbances such as delirium and acute cognitive dysfunction are well established and extensively investigated, the relationship between these transient early postoperative cognitive disturbances and long-term cognitive and functional decline in patients with established dementia is not well understood and deserves to be investigated [17,18].

Because several closely related terms are discussed in this narrative review, we define them here, following current consensus nomenclature, and use them consistently throughout. Postoperative delirium is an acute, fluctuating disturbance of attention. Delayed neurocognitive recovery is objective cognitive decline detected within 30 days after surgery in a patient without delirium. Postoperative neurocognitive disorder is cognitive decline persisting over 30 days and up to 12 months. Cognitive decline is used generically for any measurable reduction in cognitive performance, flagged as such where a more specific term does not apply. Functional decline refers to reduced ability to perform daily activities, distinct from cognitive performance [12]. Dementia progression denotes a sustained worsening of the underlying disease. In addition, dementia is a heterogeneous group of neurodegenerative and vascular disorders, not a single entity. Alzheimer’s disease, vascular dementia, Lewy body dementia, and frontotemporal dementia differ in neuronal degeneration patterns, vascular involvement, autonomic dysfunction, and neurotransmitter abnormalities that may shape susceptibility to specific perioperative stressors. For instance, patients with significant cerebrovascular disease may be more vulnerable to haemodynamic disturbances, while autonomic dysfunction and attentional fluctuations may be particularly relevant in Lewy body dementia. Disease severity, frailty, vascular burden, and baseline function may further modify perioperative risk. The mechanisms should be viewed as potentially shared vulnerability pathways rather than uniform across dementia subtypes.

Despite growing concern regarding perioperative neurocognitive disorders in patients with dementia, the current evidence base is scarce due to substantial methodological challenges. Much of the available literature is observational and heterogeneous in design and frequently excludes patients with established dementia. Separating the effects of anaesthesia, surgical stress, underlying frailty, and pre-existing neurodegenerative progression continues to pose challenges. Consequently, the causal relationship between perioperative exposures and long-term cognitive decline has not been definitively established.

The relationship between anaesthesia, surgery, and long-term dementia progression remains controversial. While some studies suggest an association between perioperative exposure and adverse neurocognitive outcomes, others have not demonstrated significant long-term cognitive effects after adjustment for age, frailty, vascular disease, and baseline cognitive impairment.

This narrative review was based on a structured literature search conducted in PubMed/MEDLINE, Scopus, ScienceDirect, and Google Scholar until July 2026 to identify studies examining the long-term cognitive and functional outcomes of anesthesia and surgery in patients with dementia. The search included combinations of the following keywords where applicable: dementia, Alzheimer’s disease, anesthesia, anaesthesia, surgery, perioperative neurocognitive disorders, postoperative delirium, postoperative cognitive dysfunction, cognitive decline, functional outcomes, and long-term outcomes. Original research articles, systematic reviews, meta-analyses, and relevant clinical guidelines published in English were considered. Studies focusing on adult patients with pre-existing dementia and reporting perioperative cognitive or functional outcomes were included, whereas studies involving pediatric populations, non-surgical settings, or lacking relevant outcome data were excluded. Additional relevant publications were identified through manual screening of the reference lists of selected articles. This narrative review aims to provide a synthesis of the current evidence based on long-term cognitive, functional, and neurobiological consequences of anaesthesia and surgery in patients with established dementia. Although this review discusses dementia as a broad clinical entity, it is important to recognize that Alzheimer’s disease, vascular dementia, Lewy body dementia, and frontotemporal dementia differ in their underlying pathophysiology and may vary in their susceptibility to perioperative complications. The mechanistic pathways discussed in this narrative review represent shared biological processes that may contribute to perioperative vulnerability across dementia syndromes while acknowledging that their relative importance may differ between dementia subtypes.

The central question addressed in this review is whether perioperative exposure to surgery and anaesthesia alters the long-term cognitive and functional outcomes of patients with dementia. These outcomes indicate increased perioperative vulnerability but do not, in themselves, demonstrate accelerated progression of the underlying neurodegenerative disease. Accordingly, this review considers two related but distinct dimensions of perioperative risk: first, the susceptibility of patients with dementia to acute and longer-term postoperative complications and, second, the more specific and currently unresolved question of whether surgery or anaesthesia independently changes the natural trajectory of dementia or accelerates neurodegeneration. This distinction provides the conceptual framework for interpreting the mechanistic and clinical evidence discussed throughout the review.

## 2. Dementia as a State of Increased Neurocognitive Vulnerability

Dementia is no longer regarded simply as the pathological manifestation of cognitive decline but rather as a complex neurobiological state characterized by decreased tolerance to physiological stress and the reduced capacity for cerebral homeostasis [19,20]. While the underlying mechanisms differ between dementia subtypes, several biological processes that increase perioperative vulnerability are shared across these conditions. As neurodegeneration progresses, structural and functional alterations accumulate, affecting the brain’s ability to maintain homeostasis during physiological stress [21,22].

Experimental studies show that neuroinflammation, blood–brain barrier dysfunction, synaptic dysfunction, altered neurotransmission, cerebrovascular dysregulation, and metabolic stress can impair neuronal function, and clinical studies in older surgical patients link these processes to postoperative delirium and other perioperative neurocognitive disorders [23]. However, direct evidence that they cause accelerated dementia progression after surgery is lacking. Biological plausibility should not be mistaken for clinical causality: these mechanisms are best viewed as potential contributors to perioperative vulnerability, not established pathways of dementia progression.

From the clinical perspective, the reduced neurophysiological resilience associated with dementia becomes particularly relevant in the perioperative period. Surgical trauma, anaesthetic exposure, systemic inflammation, hemodynamic stress, metabolic derangements, and perioperative hypoxia present considerable physiological demands on the central nervous system [13,24]. Compared with cognitively intact individuals, perioperative stressors may have a greater neurological impact in patients with dementia because pre-existing neurodegenerative changes limit compensatory responses [11,25]. However, increased biological susceptibility does not necessarily imply inevitable postoperative cognitive decline. Considerable interindividual variability exists, and many patients with dementia tolerate surgical procedures without measurable long-term deterioration [26].

**Figure 1:**
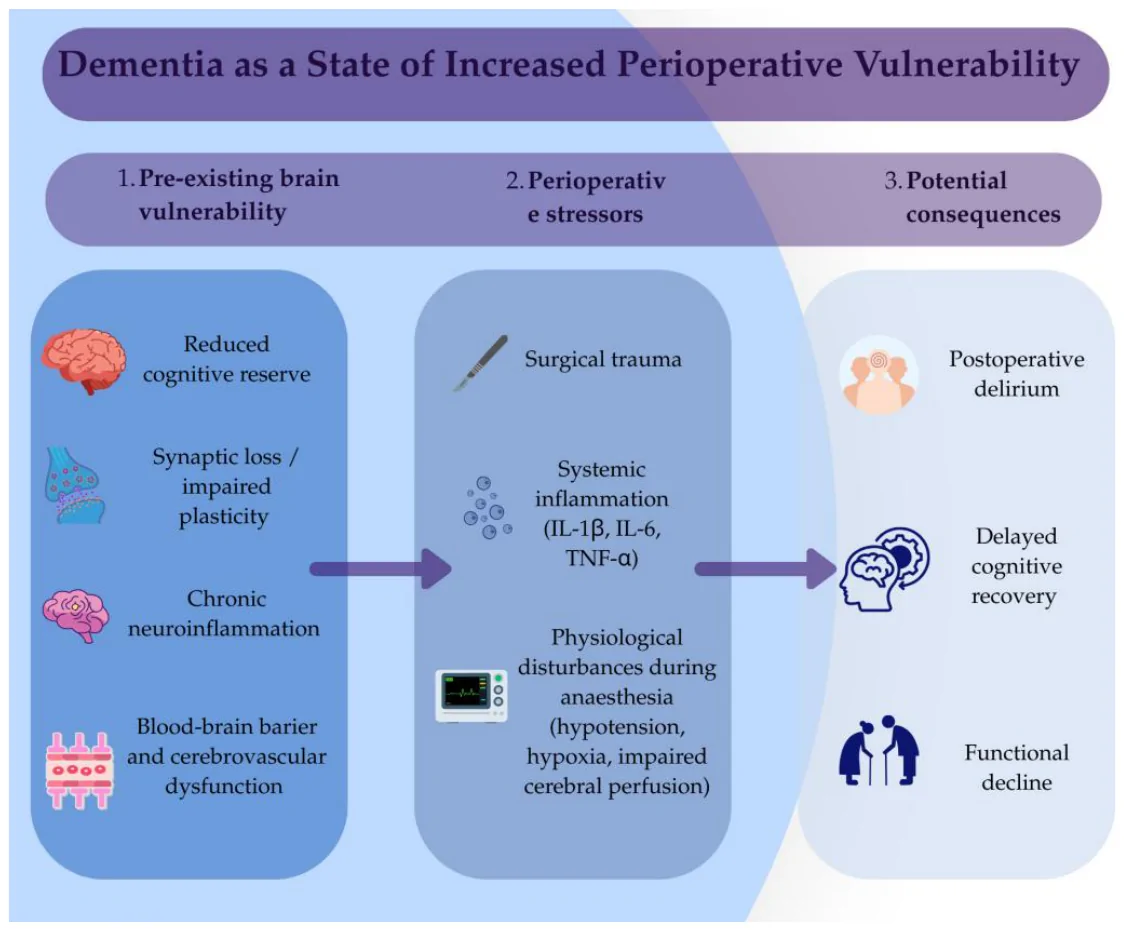
Dementia as a state of increased perioperative vulnerability. This figure illustrates the concept of dementia in which pre-existing alterations in brain structure and function increase susceptibility to perioperative stressors and adverse postoperative outcomes. Reduced cognitive reserve, synaptic loss and impaired plasticity, chronic neuroinflammation, and blood–brain barrier and cerebrovascular dysfunction contribute to an already vulnerable neural environment. During the perioperative period, surgical trauma, systemic inflammatory responses (including increased IL-1β, IL-6, and TNF-α), and physiological disturbances associated with anaesthesia (such as hypotension, hypoxia, and impaired cerebral perfusion) may further challenge cerebral homeostasis. These interactions can lead to postoperative complications, including delirium, delayed cognitive recovery, and functional decline. The figure highlights the bidirectional relationship between baseline neurocognitive vulnerability and perioperative insults, emphasizing the need for tailored perioperative strategies in patients with dementia. Figure created by the authors using Canva Pro (Canva Pty Ltd., Sydney, Australia) under a valid license.

These neurobiological mechanisms provide a plausible framework for increased perioperative susceptibility in dementia, but direct clinical evidence linking these processes to accelerated long-term decline after surgery requires further investigation. Much of the mechanistic understanding derives from experimental and translational studies, whereas human studies are frequently confounded by advanced age, frailty, comorbidity burden, and baseline cognitive impairment. Collectively, these mechanisms provide a biologically plausible framework linking dementia-related neuropathology to adverse perioperative neurocognitive outcomes (Figure 1) [27,28].

### 2.1. Cognitive Reserve and Brain Resilience

Cognitive reserve is an active functional construct that reflects the efficiency, flexibility, and adaptability of neural networks. It provides a useful framework for understanding why individuals with similar neuropathological changes may exhibit markedly different clinical outcomes. Cognitive reserve contributes to the brain’s resilience, its capacity to resist or recover from acute insults, including perioperative stressors, by enabling compensation for structural brain damage through efficient neural network utilization and adaptive cognitive strategies. It is shaped by lifelong experiences, including education, occupational attainment, intellectual enrichment, and social engagement [29,30].

In dementia, this reserve progressively declines. Neuronal loss, synaptic degeneration, and disruption of large-scale cortical networks reduce the efficiency of information processing and limit the brain’s ability to compensate for additional physiological stress [31,32]. Consequently, perioperative disturbances that may be well tolerated by cognitively healthy individuals can precipitate delirium, delayed cognitive recovery, or functional decline in patients with dementia.

Reduced cognitive reserve also limits recovery following surgery. Surgical trauma, inflammation, anaesthesia, haemodynamic instability, and metabolic disturbances place additional demands on already compromised neural networks, leaving little capacity for adaptation or repair [33,34,35,36,37,38,39,40,41].

### 2.2. Neurobiological Mechanisms of Perioperative Vulnerability

#### 2.2.1. Synaptic Dysfunction and Impaired Plasticity

Synaptic degeneration is a hallmark of dementia and one of the strongest pathological correlates of cognitive impairment [42]. Progressive loss of synapses disrupts communication between cortical and subcortical networks, while impaired synaptic plasticity limits long-term potentiation, neuronal remodelling, and adaptive strengthening of neural circuits [43,44,45].

These abnormalities reduce the brain’s capacity to recover from perioperative stress and may contribute to postoperative delirium, delayed cognitive recovery, and prolonged functional impairment, particularly after major surgery or postoperative complications [46,47]. Although this concept is supported by experimental and biomarker studies, direct evidence in surgical patients with dementia remains limited.

#### 2.2.2. Neuroinflammation and Blood–Brain Barrier Dysfunction

Chronic neuroinflammation is increasingly recognized as a central feature of neurodegenerative disease. Persistent activation of microglia and astrocytes promotes the release of pro-inflammatory cytokines, chemokines, reactive oxygen species, and other neurotoxic mediators, contributing to synaptic dysfunction and progressive neuronal injury [48,49,50,51,52].

At the same time, disruption of the blood–brain barrier may facilitate the entry of circulating inflammatory mediators into the brain during perioperative stress [53,54,55]. This increased permeability may amplify central neuroinflammatory responses and contribute to postoperative neurocognitive disorders, particularly in patients with advanced neurodegeneration or cerebrovascular disease [1,52].

Although experimental evidence strongly supports the involvement of neuroinflammation and blood–brain barrier dysfunction, their precise contribution to long-term postoperative cognitive decline in humans remains incompletely understood [46,56,57].

#### 2.2.3. Neurotransmitter Dysfunction

Dementia is associated with widespread disturbances in cholinergic, glutamatergic, GABAergic, and monoaminergic neurotransmission that contribute to impaired attention, memory, and executive function [58,59,60,61,62,63,64,65,66,67,68]. Among these systems, cholinergic dysfunction appears particularly relevant in the perioperative setting because cholinergic pathways play an essential role in attention, arousal, and cognitive stability. Anaesthetic agents and medications with anticholinergic properties may further impair already compromised neurotransmission, increasing susceptibility to postoperative delirium and delayed cognitive recovery [17,63,64,65]. Alterations in glutamatergic and GABAergic signalling may further modify responses to anaesthetic agents, as many commonly used drugs act through these pathways [66,67,68]. In the setting of pre-existing neurodegeneration, such pharmacological effects may produce exaggerated cognitive and behavioural responses. However, direct clinical evidence linking perioperative neurotransmitter disturbances to long-term cognitive decline remains limited [46].

Reduced cognitive reserve, impaired synaptic plasticity, chronic neuroinflammation, blood–brain barrier dysfunction, and neurotransmitter abnormalities do not act independently. Rather, they interact to reduce cerebral resilience and increase susceptibility to perioperative stress. This integrated model provides a biologically plausible explanation for the increased incidence of postoperative delirium, delayed cognitive recovery, and functional decline observed in patients with dementia. Consequently, current evidence supports a multifactorial model of perioperative vulnerability rather than a direct causal relationship between anaesthesia, surgery, and accelerated neurodegeneration [27,28].

## 3. Perioperative Stressors Affecting Long-Term Outcomes

Perioperative stressors associated with surgery and anaesthesia may influence long-term neurological outcomes through interacting inflammatory, metabolic, vascular, and haemodynamic mechanisms. Although these physiological perturbations occur in all surgical patients, their consequences are likely to be amplified in individuals with pre-existing neurodegenerative disease because of reduced neurophysiological resilience and impaired cerebral homeostasis [11].

### 3.1. Surgical Stress and Systemic Inflammation

Surgical trauma initiates a coordinated systemic stress response characterised by activation of the innate immune system and rapid release of pro-inflammatory mediators. Tissue injury triggers immune cascades that extend beyond the surgical site into the systemic circulation, increasing concentrations of cytokines, chemokines, and acute-phase reactants [69]. Anaesthesia and surgery together may propagate these inflammatory signals to the central nervous system, where they can amplify neuroinflammatory responses and contribute to neuronal dysfunction, postoperative delirium, and longer-lasting neurocognitive impairment [70,71].

Although this inflammatory response is essential for tissue repair and wound healing, excessive or prolonged immune activation has been associated with perioperative neurocognitive disorders and less favourable neurological outcomes [24]. However, considerable interindividual variability exists, indicating that perioperative inflammation alone is unlikely to determine postoperative cognitive trajectories [28]. Peripheral cytokines may influence the central nervous system through humoral pathways, afferent neural signalling, and regions of impaired blood–brain barrier integrity [72]. Subsequent activation of microglia and astrocytes disrupts synaptic communication and neuronal plasticity, processes that are fundamental for normal cognitive function [73,74].

In patients with dementia, these inflammatory responses may be further amplified by pre-existing neurodegenerative pathology, increasing susceptibility to postoperative neurocognitive complications [75,76,77,78]. Experimental and clinical evidence also suggests an association between perioperative neuroinflammation, postoperative delirium, and subsequent cognitive deterioration, although the causal relationship with long-term dementia progression remains incompletely established [24,79]. Clinically, exaggerated inflammatory responses have been linked to prolonged hospitalization, delayed functional recovery, greater postoperative dependency, and higher rates of postoperative delirium, particularly in patients with reduced neurocognitive reserve [17,80,81,82]. Nevertheless, current evidence supports a multifactorial model in which inflammation interacts with vascular dysfunction, frailty, baseline neurodegeneration, and postoperative complications to determine long-term neurological outcomes [11,13,24].

### 3.2. Physiological Disturbances During Anesthesia and Surgery

Besides triggering an inflammatory response, surgery and anaesthesia produce several physiological changes that may affect brain function [83]. General anaesthesia influences cardiovascular regulation, respiration, thermoregulation, and metabolic homeostasis through both the pharmacological effects of anaesthetic agents and the physiological response to surgery [84]. Although modern anaesthetic techniques and monitoring have improved patient safety, disturbances in physiological homeostasis remain common during induction, maintenance, and emergence from anaesthesia [85].

Anaesthetic agents may alter cerebral autoregulation, impair gas exchange, and influence glucose metabolism and mitochondrial function [86,87]. In addition, vasodilation, myocardial depression, and reduced sympathetic tone increase the risk of intraoperative hypotension and reduced cerebral perfusion [88,89]. Changes in respiratory function, whether related to mechanical ventilation or anaesthetic drugs, may also result in hypoxaemia or hypercapnia, both of which can impair cerebral oxygen delivery [90,91].

Healthy individuals maintain cerebral homeostasis through effective autoregulation, metabolic adaptation, and cellular stress responses [92,93]. In patients with dementia, these protective mechanisms are weakened. Neurodegeneration is associated with impaired neurovascular coupling, reduced regulation of cerebral blood flow, mitochondrial dysfunction, and increased oxidative stress, limiting the brain’s ability to tolerate physiological insults [94,95,96,97]. Consequently, even relatively mild disturbances, including hypotension, hypoxaemia, hypercapnia, hypoglycaemia, and temperature dysregulation, may increase neuronal stress and the risk of perioperative neurocognitive disorders [11,98].

Careful intraoperative management of haemodynamics, oxygenation, ventilation, temperature, and metabolic balance is therefore particularly important in patients with dementia. Although direct evidence that optimization of these physiological variables prevents long-term cognitive decline remains limited, maintaining physiological stability represents a fundamental principle of brain-protective anaesthesia [99,100].

Perioperative physiological disturbances may affect the vulnerable brain through several biologically plausible pathways, including impaired cerebral perfusion, hypoxia, metabolic dysfunction, oxidative stress, neuroinflammation, and disruption of the blood–brain barrier. However, the strength of evidence differs substantially between experimental studies, studies of short-term postoperative neurocognitive outcomes, and studies examining long-term cognitive or functional trajectories in patients with established dementia. The available evidence therefore supports careful perioperative physiological management but does not establish that these disturbances accelerate the underlying neurodegenerative disease process. Table 1 summarizes the proposed mechanisms and the current evidence across these different outcome domains.

Perioperative hypotension, hypoxaemia, disturbances in carbon dioxide homeostasis, metabolic abnormalities, and temperature dysregulation represent potentially modifiable physiological stressors in patients with dementia [180]. Their effects may be amplified by reduced cerebrovascular reserve, impaired neurovascular coupling, mitochondrial dysfunction, frailty, and pre-existing neurodegenerative or vascular pathology. Nevertheless, most mechanistic evidence derives from experimental models or from populations of older surgical patients rather than from patients with established dementia. Importantly, associations with delirium, delayed neurocognitive recovery, or postoperative functional decline should not be interpreted as evidence that these perioperative disturbances accelerate the underlying neurodegenerative process. At present, individualized physiological management should therefore be considered an important component of effective perioperative care, while the effect of specific intraoperative targets on long-term dementia trajectories remains uncertain.

### 3.3. Anaesthetic Agents and Neurotoxicity

The possibility that anaesthetic agents may contribute to long-term cognitive decline has attracted considerable attention over the past two decades. Experimental studies have shown that several commonly used anaesthetic agents can influence neuronal survival, synaptic plasticity, neuroinflammation, amyloid-β metabolism, and tau phosphorylation [183,184,185]. These findings have raised concerns that anaesthesia might accelerate neurodegenerative processes in susceptible individuals.

Most evidence supporting anaesthetic neurotoxicity, however, comes from animal models or cell culture studies using drug concentrations and exposure times that differ substantially from routine clinical practice [186,187]. Translating these findings to older surgical patients has therefore been challenging. Human studies are complicated by numerous confounding factors, including age, frailty, vascular disease, baseline cognitive impairment, surgical stress, and postoperative complications.

Experimental studies have shown that volatile anaesthetic agents can increase amyloid precursor protein processing, amyloid-β accumulation, tau phosphorylation, and neuroinflammatory activity under laboratory conditions [188,189,190,191]. These findings have raised the hypothesis that inhalational anaesthesia might influence Alzheimer’s disease-related pathology. However, clinical evidence has not consistently supported these experimental observations. Human observational studies, systematic reviews, and meta-analyses have produced mixed results, and there is currently no convincing evidence that exposure to volatile anaesthetics alone causes long-term dementia progression in clinical practice [192,193,194]. Intravenous anaesthetic agents appear to have different biological effects. Propofol has demonstrated anti-inflammatory and antioxidant properties in several experimental studies and may attenuate some neuroinflammatory responses associated with surgery [195,196]. Nevertheless, propofol also acts on neuronal signalling pathways, and current clinical evidence does not clearly demonstrate superiority over volatile anaesthesia in preventing long-term cognitive decline [197].

Other perioperative medications may also influence postoperative neurological outcomes. Benzodiazepines, anticholinergic drugs, and excessive opioid administration have all been associated with an increased risk of postoperative delirium, particularly in older adults and patients with dementia [17,65,198]. Because postoperative delirium is associated with poorer long-term cognitive and functional outcomes, careful selection and dosing of these medications remain important components of perioperative care.

Overall, current evidence suggests that postoperative neurological outcomes are determined more by the combined effects of surgical stress, inflammation, physiological instability, patient frailty, and baseline neurodegeneration than by the direct neurotoxic effects of individual anaesthetic agents [13,197,198,199]. Consequently, modern perioperative strategies should focus on optimizing the entire perioperative pathway rather than selecting one anaesthetic drug over another solely to prevent long-term cognitive decline.

## 4. Clinical Evidence on Long-Term Cognitive and Functional Outcomes

### 4.1. Long-Term Cognitive Trajectories After Surgery

The long-term cognitive effects of anaesthesia and surgery in patients with pre-existing dementia remain an important but unresolved clinical question. Patients with dementia have reduced cognitive reserve and increased susceptibility to perioperative neurocognitive disorders (PNDs), yet current evidence does not demonstrate that anaesthesia or surgery alone accelerates neurodegenerative progression independently of other patient- and disease-related factors [46,149].

Observational and longitudinal studies suggest that some patients experience persistent cognitive decline after major surgery, particularly when postoperative recovery is complicated by delirium, systemic inflammation, prolonged hospitalization, or physiological instability [66,150]. Declines are most commonly observed in memory, executive function, processing speed, and global cognition and appear more frequent after major cardiac, orthopaedic, and vascular procedures, which are associated with substantial inflammatory and haemodynamic stress [151,152,153]. However, the magnitude of these changes is generally modest compared with the effects of advanced age, frailty, cerebrovascular disease, and the underlying neurodegenerative process [35,153,154].

Baseline cognitive impairment is one of the strongest predictors of adverse postoperative outcomes. Compared with cognitively healthy individuals, patients with dementia are more likely to develop postoperative delirium, delayed neurocognitive recovery, prolonged hospitalization, and subsequent loss of independence [155,156,157]. These findings support the concept that pre-existing brain vulnerability, rather than perioperative exposure alone, largely determines postoperative cognitive trajectories.

Despite these observations, evidence linking anaesthesia directly to long-term cognitive decline remains inconsistent. Several systematic reviews and meta-analyses have found no convincing association between anaesthetic exposure and subsequent dementia after adjustment for important confounders, including age, cardiovascular disease, frailty, cerebrovascular pathology, educational level, and baseline cognitive status [158,159,160]. Consequently, it remains difficult to distinguish postoperative cognitive decline from the natural progression of dementia.

Interpretation of the literature is further complicated by considerable methodological heterogeneity. Studies differ in patient populations, surgical procedures, anaesthetic techniques, cognitive assessment tools, diagnostic criteria, and duration of follow-up, limiting direct comparison between studies and reducing the strength of pooled conclusions [160,161,162,163]. In addition, separating the effects of anaesthesia from those of surgery is inherently difficult because surgical trauma induces systemic inflammation, neuroendocrine activation, metabolic stress, and physiological disturbances that may independently influence brain function [11,60,164]. Current evidence therefore supports a multifactorial model in which postoperative cognitive outcomes reflect the combined effects of pre-existing neurodegeneration, frailty, vascular disease, perioperative physiological disturbances, systemic inflammation, and postoperative complications rather than anaesthetic exposure alone [11,28,79].

Among perioperative complications, postoperative delirium consistently shows the strongest association with long-term cognitive deterioration. Patients who develop delirium are more likely to experience persistent cognitive impairment, poorer functional recovery, institutionalization, and increased mortality. However, whether delirium directly accelerates neurodegeneration or primarily reflects reduced baseline brain resilience remains uncertain [80,165,166].

Importantly, postoperative cognitive decline is not inevitable. Many patients with dementia recover without measurable long-term deterioration, highlighting the substantial interindividual variability in postoperative outcomes. Differences in dementia severity, cognitive reserve, frailty, comorbidity burden, perioperative management, and postoperative complications probably account for much of this variability [151,167]. Increasing evidence suggests that comprehensive perioperative care, including optimization of physiological parameters, delirium prevention, and early multidisciplinary rehabilitation, may improve postoperative recovery in this high-risk population [60,168].

Overall, current clinical evidence indicates that patients with dementia are at increased risk of adverse postoperative cognitive outcomes. However, a direct causal relationship between anaesthesia, surgery, and accelerated dementia progression has not been established. Long-term cognitive trajectories are more likely to reflect complex interactions between perioperative stressors and pre-existing neurodegenerative vulnerability than the effects of anaesthesia alone. Future prospective studies that specifically include patients with established dementia are needed to identify modifiable perioperative risk factors and clarify long-term cognitive outcomes [158,159,160,161,162,163,164,165,166,167,168].

An important distinction must be made between adverse postoperative outcomes and progression of the underlying neurodegenerative disease. Postoperative delirium, delayed neurocognitive recovery, persistent cognitive decline, functional dependence, institutionalization, and mortality represent clinically important but distinct outcomes. Their occurrence does not necessarily indicate that surgery or anaesthesia has accelerated the biological progression of dementia. A patient may experience delayed cognitive or functional recovery after surgery because of delirium, pain, inflammation, immobility, infection, or other postoperative complications and subsequently return toward the preoperative trajectory. Conversely, a persistent change in cognitive trajectory may suggest a more sustained effect, although establishing such a change requires longitudinal assessment and appropriate non-surgical comparison groups. Therefore, postoperative cognitive or functional deterioration should not be interpreted automatically as evidence of accelerated neurodegeneration. A sustained downward shift in cognitive or functional trajectory can only be confirmed through longitudinal assessment comparing postoperative performance with a well-characterized preoperative baseline, ideally alongside a non-surgical reference group. Thus, “long-term decline” and “dementia progression” should be reserved for findings that persist beyond the expected recovery period and, preferably, deviate from the individual’s expected disease trajectory.

### 4.2. Functional Outcomes and Loss of Independence

Beyond cognitive decline, loss of functional independence is one of the most important long-term consequences of surgery in patients with dementia [166]. Functional outcomes directly affect quality of life, caregiver burden, rehabilitation potential, and the need for institutional care. For many older adults, maintaining independence in daily activities may be as important as preserving cognitive function [169,170].

Patients with dementia often have reduced physiological reserve before surgery because of frailty, sarcopenia, malnutrition, impaired mobility, and reduced ability to perform activities of daily living (ADLs) [172,173]. These pre-existing vulnerabilities may limit postoperative recovery. Compared with cognitively healthy patients, individuals with dementia are more likely to experience delayed mobilization, prolonged physical disability, and greater dependence after major surgery [154,171,174].

Several prospective studies have shown that pre-existing cognitive impairment is strongly associated with poorer postoperative functional recovery. Older adults with impaired cognition are more likely to experience a decline in ADLs after surgery, particularly following cardiac and orthopaedic procedures. Patients who develop postoperative delirium are at especially high risk of losing independence and often require assistance with dressing, bathing, medication management, and mobility during long-term follow-up [175,176,177].

Hip fracture surgery provides one of the best clinical models for studying postoperative functional recovery in patients with dementia. Although surgical repair is often successful, recovery of pre-fracture function is frequently incomplete. Many patients remain unable to regain their previous level of mobility or independence several months after surgery. Persistent gait impairment, reduced participation in rehabilitation, prolonged immobility, recurrent hospitalization, and fear of falling all contribute to sustained disability [178,179,180,181,182,183,184,185,186,187,188,189].

Postoperative delirium appears to play a central role in functional recovery. Numerous studies and meta-analyses have consistently associated delirium with poorer recovery of physical function, greater dependence in activities of daily living, reduced quality of life, and increased rates of institutionalization [18,176,190]. Delirium may delay recovery by limiting early mobilization, reducing participation in rehabilitation, impairing communication, worsening nutritional intake, disrupting sleep, and increasing the risk of secondary complications such as falls and infections. These effects may be particularly pronounced in patients with dementia because baseline cognitive impairment already limits adaptive capacity and functional reserve [17,80,138].

Functional decline also has important consequences beyond the individual patient. Persistent loss of independence increases caregiver burden, healthcare utilization, and the need for long-term support services [191,192]. Patients with prolonged postoperative disability frequently require home assistance, rehabilitation programmes, or permanent institutional care. Long-term care placement is particularly common after postoperative delirium, prolonged immobility, or major postoperative complications [163,193].

Compared with cognitively intact older adults, patients with dementia are substantially more likely to be discharged to nursing homes or long-term care facilities rather than returning home after surgery [18,194]. This transition often reflects the combined effects of worsening cognition, impaired mobility, reduced self-care capacity, and increasing caregiver burden rather than surgical complications alone.

Importantly, postoperative functional decline is not inevitable. Recovery varies considerably between individuals and depends on dementia severity, frailty, nutritional status, comorbidities, social support, and access to rehabilitation. Many patients achieve meaningful functional recovery, particularly when perioperative complications are prevented and rehabilitation begins early. Increasing evidence supports multidisciplinary perioperative care that combines geriatric assessment, delirium prevention, nutritional optimization, cognitive support, early mobilization, and individualized rehabilitation to improve long-term functional outcomes [99,138,195].

Despite growing recognition of functional outcomes, important knowledge gaps remain. Many studies focus primarily on mortality or cognitive performance and pay less attention to quality of life, independence, and long-term functional recovery. Furthermore, distinguishing postoperative functional decline from the natural progression of dementia remains difficult, particularly in studies with limited follow-up or inadequate baseline functional assessment. Future prospective studies should therefore include standardized measures of functional status alongside cognitive outcomes to better define recovery trajectories and identify modifiable perioperative risk factors [81,99].

### 4.3. Postoperative Delirium as a Mediator of Long-Term Decline

Postoperative delirium is the most important perioperative complication in patients with dementia and represents the strongest clinical predictor of adverse long-term cognitive and functional outcomes. Although delirium is traditionally considered an acute and reversible syndrome, increasing evidence indicates that its consequences frequently extend beyond the immediate postoperative period, particularly in individuals with pre-existing cognitive impairment [17,150].

Patients with dementia are especially susceptible to postoperative delirium because pre-existing neurodegeneration reduces the brain’s capacity to compensate for perioperative physiological stress. Rather than acting as an isolated cause of neurological deterioration, surgery and anaesthesia may precipitate delirium by overwhelming an already vulnerable brain [196,197,198].

The clinical significance of postoperative delirium extends well beyond transient confusion. Numerous prospective studies have consistently associated delirium with prolonged hospitalization, postoperative complications, persistent cognitive impairment, functional decline, institutionalization, and increased mortality [18,199]. Longitudinal studies further demonstrate that patients who develop postoperative delirium are more likely to experience sustained deficits in memory, attention, executive function, and global cognition months or years after surgery [17,200]. These effects appear particularly important in patients with dementia, in whom even modest additional cognitive decline may substantially reduce independence.

Whether delirium directly accelerates neurodegeneration or primarily reflects reduced baseline brain resilience remains uncertain. Both mechanisms are biologically plausible and are not mutually exclusive. Delirium may contribute to poorer long-term outcomes by promoting prolonged neuroinflammatory responses, immobility, sleep disruption, inadequate nutritional intake, reduced participation in rehabilitation, and secondary medical complications. Equally, it may represent a clinical marker of advanced neurodegeneration, frailty, cerebrovascular disease, and limited cognitive reserve [16,52,198].

The consequences of delirium extend beyond cognition. Patients who experience postoperative delirium are more likely to have delayed mobilization, impaired rehabilitation, loss of independence in activities of daily living, and permanent institutionalization, particularly after major orthopaedic surgery such as hip fracture repair [175,190,201,202]. These outcomes substantially increase caregiver burden, healthcare utilization, and long-term social care needs.

An important clinical challenge is distinguishing persistent delirium from progression of the underlying dementia. Ongoing confusion, impaired attention, reduced mobility, and functional decline after surgery may reflect unresolved delirium, progression of neurodegenerative disease, or both. Because delirium is potentially reversible, careful postoperative assessment is essential to identify treatable causes and avoid premature assumptions regarding irreversible cognitive deterioration [203,204].

Unlike many other perioperative risk factors, postoperative delirium is at least partly preventable. Multicomponent non-pharmacological interventions, including orientation strategies, early mobilization, sleep promotion, adequate hydration, effective analgesia, medication review, optimization of vision and hearing, and avoidance of unnecessary sedatives or anticholinergic drugs, reduce the incidence and severity of delirium and remain central components of perioperative care [205,206,207].

Postoperative delirium may represent a potential intermediary between perioperative stress and subsequent cognitive and functional deterioration, but its precise causal role remains uncertain. Delirium may contribute directly to adverse outcomes through prolonged neuroinflammatory activation, sleep disruption, immobility, inadequate nutrition, reduced participation in rehabilitation, and secondary complications [208]. Alternatively, delirium may primarily function as a clinical marker of pre-existing brain vulnerability, reflecting advanced neurodegeneration, frailty, cerebrovascular disease, reduced cognitive reserve, or limited physiological resilience. These interpretations are not mutually exclusive. Current evidence does not establish that delirium independently accelerates the underlying neurodegenerative process, and longitudinal studies incorporating appropriate measures of baseline vulnerability are needed to determine whether delirium represents a causal pathway, a marker of vulnerability, or both.

**Figure 2:**
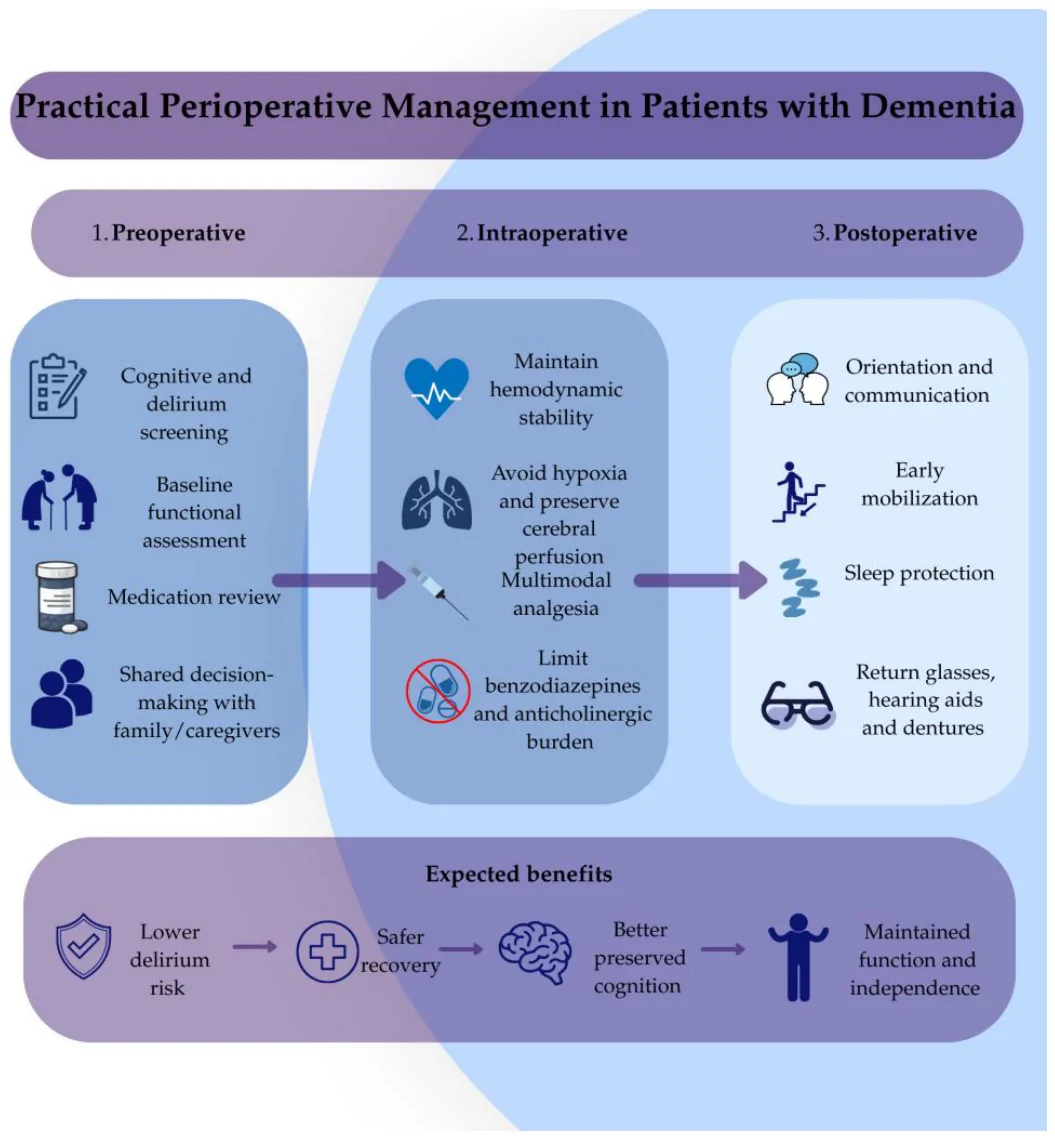
Practical perioperative management in patients with dementia: strategies across the surgical care continuum to reduce complications and preserve function. This figure summarizes practical, evidence-informed strategies for the perioperative management of patients with dementia across three key phases: preoperative, intraoperative, and postoperative care. During the preoperative phase, comprehensive assessment should include cognitive and delirium screening, evaluation of baseline functional status, medication review, and shared decision-making involving patients, families, and caregivers. These measures help identify individual risks and establish patient-centered care goals. During the intraoperative phase, strategies focus on maintaining cerebral and physiological stability, including avoidance of hypoxia, preservation of cerebral perfusion, multimodal analgesia, and minimization of medications associated with cognitive adverse effects, such as benzodiazepines and anticholinergic agents. During the postoperative phase, prevention and management of cognitive and functional complications involve maintaining orientation and effective communication, promoting early mobilization, protecting sleep, and ensuring the prompt return of sensory aids. Implementation of these integrated interventions may reduce delirium risk, support safer recovery, improve preservation of cognition, and promote maintenance of functional independence in patients with dementia undergoing surgery. Figure created by the authors using Canva Pro (Canva Pty Ltd., Sydney, Australia) under a valid license.

Postoperative delirium should therefore be viewed as both a marker of reduced brain resilience and a potentially modifiable contributor to poor postoperative recovery. While current evidence does not establish that delirium independently accelerates dementia progression, its consistent association with cognitive decline, loss of functional independence, institutionalization, and mortality makes prevention and early recognition key priorities in the perioperative management of patients with dementia [17,18,199,200,201,202,203,204,205,206,207]. Practical perioperative management in patients with dementia is summarized in Figure 2.

### 4.4. Mortality and Global Prognosis After Surgery

Patients with dementia have a substantially higher risk of adverse postoperative outcomes than cognitively healthy older adults. In addition to cognitive and functional decline, they experience higher mortality, prolonged hospitalization, postoperative complications, hospital readmission, and loss of long-term independence. These outcomes primarily reflect reduced physiological reserve, frailty, multimorbidity, and impaired recovery capacity rather than neurological impairment alone [209,210,211].

Large observational studies consistently demonstrate increased postoperative mortality among patients with dementia undergoing orthopaedic, cardiac, abdominal, and emergency surgery [212,213]. Mortality is particularly high after emergency procedures, where delayed presentation, greater physiological stress, and limited opportunities for preoperative optimization contribute to poorer outcomes. Advanced dementia, frailty, malnutrition, cardiovascular disease, impaired mobility, and multiple comorbidities further increase perioperative risk [211,212,213,214].

The increased risk extends beyond the immediate postoperative period. Population-based studies report higher mortality at 30 days, 90 days, and one year after surgery, together with greater use of intensive postoperative interventions, including prolonged mechanical ventilation, feeding tube placement, repeated hospitalization, and institutional care [179,215,216,217,218,219,220]. These findings indicate that postoperative recovery in patients with dementia frequently continues long after hospital discharge.

Postoperative complications further contribute to poor prognosis. Compared with cognitively intact patients, individuals with dementia are more likely to develop postoperative delirium, pneumonia, urinary tract infections, respiratory complications, falls, pressure injuries, prolonged immobility, and hospital-acquired functional decline [47,221]. Communication difficulties, reduced participation in rehabilitation, and delayed recognition of complications may further compromise recovery, creating a cycle of progressive disability during hospitalization.

Hospital readmission is another indicator of impaired recovery. Common causes include infection, recurrent falls, medication-related complications, nutritional decline, worsening functional dependence, and inadequate social support after discharge [222,223]. These events often necessitate prolonged rehabilitation and increase healthcare utilization well beyond the initial surgical episode.

Long-term prognosis is strongly influenced by postoperative delirium and functional decline. Patients who develop delirium are more likely to experience prolonged hospitalization, institutionalization, and increased mortality. Delayed mobilization, impaired nutritional status, dehydration, aspiration, and secondary infections may further compromise recovery, particularly in individuals with limited cognitive and physiological reserve [18,165].

Loss of independence is a major determinant of overall prognosis. Many patients who lived independently before surgery require rehabilitation facilities or permanent nursing home placement because they fail to regain their previous level of mobility or self-care. Consequently, postoperative outcomes should be evaluated not only by survival but also by preservation of functional independence, quality of life, and caregiver burden [224,225].

Despite the increased risks associated with dementia, postoperative outcomes remain highly heterogeneous. Recovery depends on dementia severity, frailty, nutritional status, cardiovascular disease, baseline functional capacity, urgency of surgery, postoperative complications, and the quality of perioperative care. Patients with early-stage dementia undergoing elective surgery generally have substantially better outcomes than those with advanced dementia requiring emergency procedures [226,227,228]. Comprehensive perioperative management—including geriatric assessment, frailty evaluation, delirium prevention, nutritional optimization, individualized anaesthetic care, early mobilization, and structured rehabilitation—may improve recovery and support more informed perioperative decision-making [5,229].

### 4.5. Current Evidence, Limitations, and Knowledge Gaps

Despite growing interest in the long-term effects of anaesthesia and surgery in patients with dementia, definitive conclusions regarding causality remain elusive. Although numerous studies report associations between perioperative exposure and adverse cognitive or functional outcomes, the available evidence is limited by substantial methodological heterogeneity, inconsistent outcome definitions, and the complex interplay between neurodegeneration, ageing, frailty, and systemic illness. Consequently, distinguishing the independent contribution of anaesthesia and surgery from the natural progression of dementia remains one of the greatest challenges in perioperative neurocognitive research [230,231].

Most available evidence is derived from observational or retrospective studies, making it difficult to distinguish causal relationships from associations. Compared with cognitively healthy controls, patients with dementia differ substantially in age, frailty, comorbidity burden, functional status, medication use, and cerebrovascular disease, all of which independently influence postoperative outcomes [230,231,232,233]. In addition, patients with advanced dementia are frequently excluded from clinical studies because of challenges related to informed consent, limited life expectancy, or the feasibility of neuropsychological assessment. Consequently, many studies predominantly include patients with mild cognitive impairment or early-stage dementia, limiting the generalizability of their findings [234,235]. This recurring conflation of mild cognitive impairment with established dementia across the literature is itself a major source of heterogeneity, and we encourage future studies to report outcomes separately for these two populations rather than as a single “cognitively impaired” group.

Interpretation is further complicated by confounding by indication. Patients requiring surgery often have cardiovascular disease, malignancy, chronic inflammation, vascular risk factors, or other serious illnesses that may themselves contribute to cognitive decline and poorer long-term outcomes. Separating the effects of surgery and anaesthesia from those of the underlying disease is therefore inherently difficult [236,237,238]. Considerable heterogeneity in surgical procedures, anaesthetic techniques, perioperative management, follow-up duration, and study design further limits direct comparison between studies. Overall, current evidence supports a multifactorial model in which postoperative outcomes reflect the combined effects of surgical stress, systemic inflammation, physiological disturbances, baseline neurodegeneration, and patient vulnerability rather than anaesthesia alone [13,239].

Assessment of cognitive outcomes represents another important limitation. Studies employ different neuropsychological tests, diagnostic criteria, assessment time points, and definitions of postoperative neurocognitive disorders. Some focus on postoperative delirium or delayed neurocognitive recovery, whereas others evaluate incident dementia years after surgery. Many also lack comprehensive preoperative cognitive assessment, making it difficult to distinguish new postoperative decline from pre-existing impairment [13,239,240,241]. Many studies also rely on a single Mini-Mental State Examination (MMSE) cut-off, commonly a score below 24, to define or exclude cognitive impairment [r]. This threshold should not be treated as a universally valid definition of cognitive impairment or dementia because MMSE performance is influenced by education, language, and cultural background, and its sensitivity varies by dementia phenotype. Similarly, differentiating postoperative cognitive decline from the natural progression of dementia remains challenging because cognitive trajectories vary according to dementia subtype, vascular burden, genetics, cognitive reserve, educational level, and comorbidity. Without carefully matched non-surgical control groups and repeated longitudinal assessments, it is difficult to determine whether surgery accelerates neurodegeneration or whether observed decline reflects the expected course of the underlying disease [11,35].

Additional bias arises from incomplete long-term follow-up. Patients with the poorest postoperative outcomes are more likely to die or become unable to participate in subsequent assessments, whereas those who recover well are more likely to remain in follow-up. This survivorship bias may underestimate the true burden of long-term cognitive and functional decline, particularly in older adults with dementia, who have high mortality and institutionalization rates [242,243]. Furthermore, dementia should not be regarded as a single disease entity. Alzheimer’s disease, vascular dementia, Lewy body dementia, and frontotemporal dementia differ substantially in their underlying pathology, cerebrovascular involvement, autonomic dysfunction, and susceptibility to delirium. Analysing these disorders as a single diagnostic category may therefore obscure important differences in perioperative vulnerability and recovery [244,245].

Addressing these limitations will require prospective, adequately powered longitudinal studies incorporating comprehensive baseline cognitive and functional assessment, standardized definitions of postoperative neurocognitive disorders, and uniform outcome measures [239,240,241]. Future research should include patients across the full spectrum of dementia severity and better characterize dementia subtype, frailty, vascular burden, and other factors influencing perioperative risk. Particular attention should be given to evaluating interventions that may improve long-term outcomes, including individualized haemodynamic management, delirium prevention, multimodal analgesia, electroencephalographic monitoring of anaesthetic depth, optimization of cerebral oxygenation, enhanced recovery pathways, and multidisciplinary geriatric co-management. Although many of these strategies improve short-term outcomes, their effects on long-term cognition and functional recovery remain uncertain. These long-term outcomes are themselves distinct constructs that should not be conflated. Mortality, institutionalization, functional independence, quality of life, caregiver burden, and cognitive performance are related but separable endpoints, each requiring its own measurement approach, and an intervention or risk factor that affects one does not necessarily affect the others; for example, a strategy that reduces delirium incidence may improve short-term functional recovery without demonstrably altering long-term cognitive trajectory or mortality.

Future studies should also evaluate outcomes that are meaningful to patients and caregivers, including functional independence, quality of life, behavioural symptoms, caregiver burden, institutionalization, and healthcare utilization, rather than focusing exclusively on neuropsychological performance, and should report these domains separately rather than as a composite “adverse outcome”. Integration of blood-based biomarkers, neuroinflammatory markers, Alzheimer’s disease biomarkers, and advanced neuroimaging may further clarify the biological mechanisms linking perioperative stress to long-term neurological outcomes and help identify patients at greatest risk [246,247,248,249,250,251,252].

Overall, current evidence indicates that patients with dementia are particularly vulnerable to adverse postoperative cognitive and functional outcomes. However, it does not support a direct causal relationship between anaesthesia and accelerated neurodegeneration. Instead, postoperative trajectories appear to reflect the cumulative effects of underlying neurodegenerative disease, ageing, frailty, comorbidity, and perioperative stress. Large multicentre prospective studies with prolonged follow-up and carefully matched non-surgical comparison groups are now needed to identify modifiable perioperative risk factors and determine which strategies can improve long-term outcomes in this vulnerable population [247,251,253]. In addition, repeated pre- and post-surgical assessments, adjustment for baseline severity and frailty, and careful handling of mortality and loss to follow-up are essential to determine whether perioperative exposure causes a sustained deviation from the underlying disease trajectory.

## 5. Perioperative Management and Future Directions

### 5.1. Preoperative Assessment and Risk Stratification

The perioperative management of patients with dementia requires a comprehensive, multidisciplinary approach that addresses cognitive vulnerability throughout the surgical pathway. Current evidence indicates that no single intervention can reliably prevent perioperative neurocognitive disorders. Instead, improved outcomes are most likely achieved through systematic risk assessment, individualized perioperative care, optimization of physiological homeostasis, postoperative delirium prevention, and coordinated multidisciplinary management.

Because patients with dementia are particularly susceptible to adverse postoperative outcomes, perioperative decision-making should begin with a careful evaluation of the anticipated benefits and risks of surgery. Shared decision-making involving patients, families, and caregivers is especially important before elective procedures, when there is sufficient time to optimize comorbidities, discuss realistic expectations, and establish patient-centred goals of care. It is important to emphasize that a diagnosis of dementia does not, in itself, equate to loss of decision-making capacity. Capacity is decision-specific and may fluctuate over time; it must be formally assessed in relation to the particular decision at hand, such as consent to a specific surgical procedure, rather than inferred from a dementia diagnosis or a cognitive screening score alone. Many patients with mild-to-moderate dementia retain the capacity to participate meaningfully in shared decision-making about their own care. Involvement of family members or legally authorized representatives should follow a formal determination that capacity for the relevant decision is impaired, in accordance with local legal and ethical frameworks, rather than proceeding automatically from the diagnosis itself. In contrast, urgent surgery often provides fewer opportunities for preoperative optimization and individualized planning [253].

Recognition of patients at increased risk of perioperative neurocognitive disorders should form part of the routine preoperative evaluation. Assessment should include baseline cognitive function, frailty, functional status, nutritional status, comorbidities, polypharmacy, previous delirium, and sensory impairment. These factors facilitate risk stratification and help identify patients who may benefit from targeted perioperative interventions and closer postoperative surveillance.

Perioperative interventions should be interpreted according to the outcomes they have actually been shown to affect. Measures such as maintaining oxygenation, haemodynamic stability, normothermia, analgesia, medication review, nutritional support, and early mobilization reflect general good perioperative care. Multicomponent delirium-prevention programmes have evidence supporting reduced delirium and select short-term complications, but evidence that any of these interventions prevent persistent cognitive decline or alter dementia trajectory remains limited. Improving short-term delirium or functional recovery should therefore not be equated with preventing long-term neurodegeneration, particularly where evidence derives mainly from older surgical populations without established dementia.

**Figure 3:**
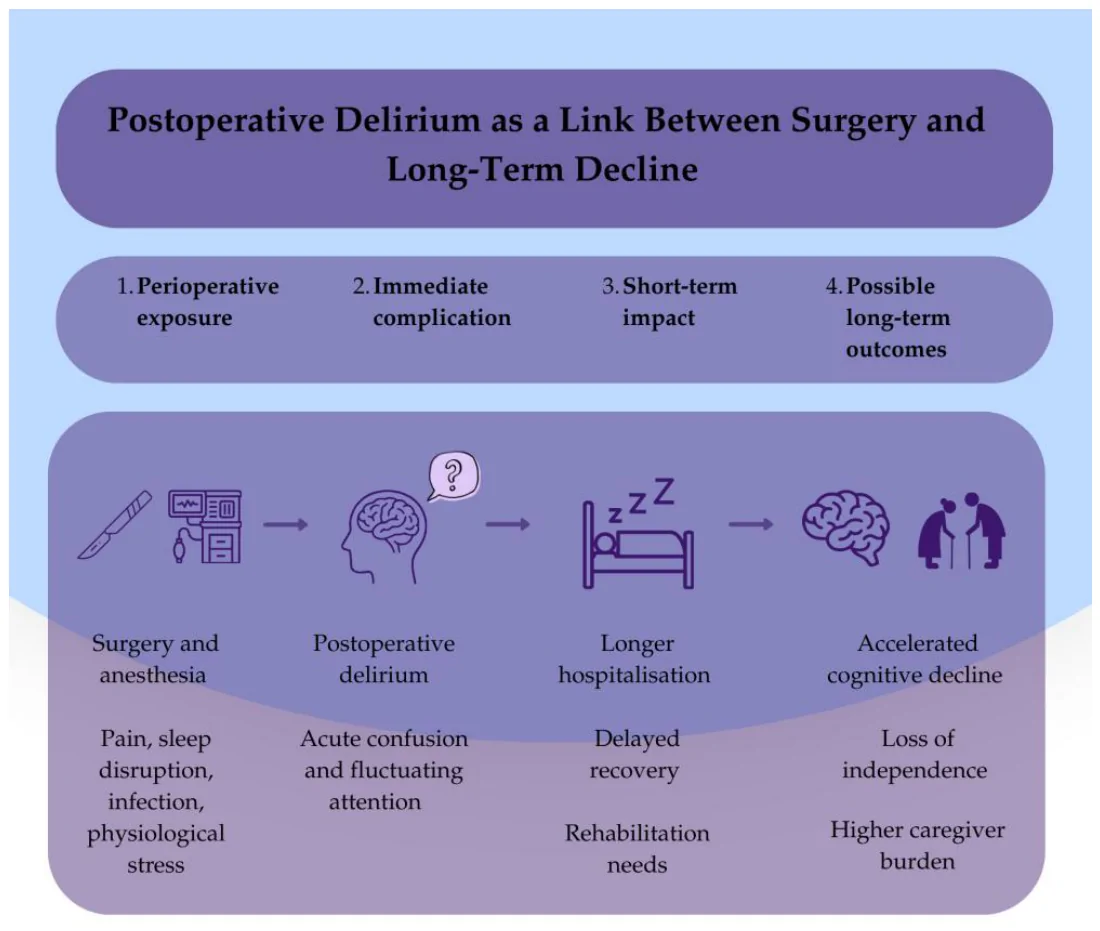
Postoperative delirium as a potential link between perioperative stress and long-term cognitive and functional decline in patients with dementia. This figure illustrates the proposed sequence through which postoperative delirium may contribute to long-term adverse outcomes after surgery. The pathway begins with perioperative exposure, including surgery, anesthesia, pain, sleep disruption, infection, and physiological stress, which increase the risk of postoperative delirium, characterized by acute confusion and fluctuating attention. Delirium is associated with short-term consequences, such as prolonged hospitalization, delayed recovery, and increased rehabilitation needs. Over time, these complications may contribute to long-term outcomes, including accelerated cognitive decline, loss of functional independence, and greater caregiver burden. The figure highlights postoperative delirium as a potential intermediary between perioperative stressors and long-term cognitive and functional deterioration. Figure created by the authors using Canva Pro (Canva Pty Ltd., Sydney, Australia) under a valid license.

Effective perioperative management also depends on close collaboration between anaesthesiologists, surgeons, geriatricians, nurses, rehabilitation specialists, patients, and caregivers. Multidisciplinary planning allows individualized perioperative strategies, improves communication across the care pathway, and supports safer recovery in this high-risk population. The key components of perioperative management across the preoperative, intraoperative, and postoperative phases are summarized in Figure 3.

### 5.2. Intraoperative Management

Patients with dementia are particularly vulnerable to physiological disturbances during anaesthesia, making individualized intraoperative management essential. Current perioperative guidelines emphasize maintaining physiological stability while minimizing factors associated with postoperative delirium and other perioperative neurocognitive disorders. Although no single intraoperative intervention has consistently improved long-term cognitive outcomes, a combination of brain-protective strategies is recommended.

Avoidance of excessive anaesthetic depth has received considerable attention as a potentially modifiable risk factor for postoperative delirium. Several professional societies, including the American Geriatrics Society, the European Society of Anaesthesiology, and the UK National Institute for Health and Care Excellence, recommend considering EEG-guided anaesthesia in patients at increased risk of delirium, and this recommendation was based predominantly on evidence regarding short-term outcomes, particularly postoperative delirium incidence, and not on evidence that EEG-guided anaesthesia prevents long-term cognitive decline or alters dementia trajectory. However, a large randomized trial found that EEG-guided anaesthetic management did not significantly reduce postoperative delirium, contrasting with earlier meta-analyses that suggested a protective effect. Given these mixed findings, EEG monitoring should be considered as part of an individualized perioperative approach rather than a proven strategy for preventing postoperative delirium [254,255,256,257,258,259,260].

The choice of anaesthetic technique has also been extensively investigated. Although experimental studies have raised concerns regarding anaesthetic-induced neurotoxicity, current clinical evidence does not demonstrate a consistent advantage of regional over general anaesthesia for preventing postoperative delirium or postoperative cognitive dysfunction. Randomized trials and meta-analyses similarly report comparable cognitive outcomes and postoperative mortality between anaesthetic techniques, suggesting that patient characteristics, surgical stress, frailty, and perioperative management are more important determinants of neurological outcome than anaesthetic modality alone [261,262,263,264,265,266,267].

EEG-based monitoring may help avoid unnecessarily deep anaesthesia and prolonged burst suppression, both of which have been associated with an increased risk of postoperative delirium, particularly in older adults with reduced cognitive reserve. Nevertheless, increasing evidence suggests that burst suppression may also reflect underlying brain vulnerability rather than excessive anaesthetic administration alone. Consequently, interpretation of EEG findings should be individualized and considered together with patient-specific risk factors [268,269,270,271,272,273,274,275,276,277,278].

Experimental evidence suggests that commonly used anaesthetic agents may influence neuronal apoptosis, mitochondrial function, oxidative stress, neuroinflammation, and tau phosphorylation. However, most supporting data derive from laboratory models, whereas clinical studies have not established a direct causal relationship between anaesthetic exposure and accelerated neurodegeneration. Similarly, where general perioperative guidelines recommend maintenance of normothermia and individualized haemodynamic management, these recommendations rest on evidence supporting general perioperative safety and reduced short-term complications, not on evidence that they prevent long-term cognitive decline specifically in patients with dementia. Current practice therefore emphasizes minimizing unnecessary anaesthetic exposure, maintaining normothermia, preserving cerebral perfusion, and considering individualized anaesthetic techniques rather than selecting specific agents solely to prevent long-term cognitive decline [279,280,281,282,283,284,285,286,287,288,289,290].

### 5.3. Postoperative Care and Delirium Prevention

Postoperative management should focus on preventing delirium, promoting functional recovery, and minimizing complications that may contribute to long-term cognitive and functional decline. Early recognition of patients at increased risk, combined with multidisciplinary postoperative care, is essential for improving outcomes in patients with dementia.

Neuropsychiatric symptoms, including agitation, anxiety, depression, sleep disturbances, apathy, and behavioural and psychological symptoms of dementia, are common after surgery and may complicate recovery independently of persistent delirium. These manifestations can impair participation in rehabilitation, prolong hospitalization, increase caregiver burden, and reduce quality of life. Prompt recognition and management of these symptoms should therefore be integrated into routine postoperative care [291,292,293,294,295,296,297,298,299,300,301,302]. Comprehensive postoperative care should incorporate evidence-based, non-pharmacological interventions that reduce delirium risk and facilitate recovery. Early mobilization, regular orientation, adequate pain control, restoration of vision and hearing through sensory aids, sleep promotion, hydration, nutritional support, physiotherapy, and comprehensive geriatric assessment have all been associated with improved postoperative outcomes. In high-risk older adults, multicomponent non-pharmacological programmes have reduced delirium incidence by up to 40% [81,303]. However, this estimate derives predominantly from broader hospitalized older adult populations rather than from surgical patients with established dementia specifically, and it should be interpreted as a relative risk reduction [81,303].

Medication management is another key component of postoperative care. Older adults are particularly susceptible to adverse drug effects, making individualized analgesic regimens and regular medication review essential. Adequate pain control should be achieved while avoiding unnecessary exposure to potentially deliriogenic medications, particularly prolonged use of benzodiazepines and antipsychotics. Current evidence does not support the routine prophylactic use of antipsychotic medication for delirium prevention. Their use should instead be reserved for carefully selected, short-term treatment of severe agitation or distress. Current evidence supports multimodal analgesia and careful balancing of analgesic efficacy against the risk of neurocognitive complications [304,305,306,307,308,309,310,311,312,313,314,315,316,317].

Family members and caregivers play an important role throughout postoperative recovery. Their participation facilitates orientation, communication, recognition of subtle cognitive changes, and implementation of non-pharmacological delirium prevention strategies. Close collaboration between patients, caregivers, and the multidisciplinary team improves continuity of care and may reduce the incidence and severity of postoperative delirium [214,306,307,308,309,310]. Despite growing evidence supporting these interventions, their routine implementation remains inconsistent because of limited resources, variable clinical practice, and the absence of standardized perioperative pathways [304,305,318,319].

Ethical considerations should also be incorporated into perioperative care for patients with dementia. Decision-making should respect patient autonomy whenever possible and involve family members or legally authorized representatives when decision-making capacity is impaired. Advance care planning, individualized assessment of surgical goals, and careful consideration of postoperative quality of life are particularly important in this population. Where temporary restrictions or additional supervision are required during hospitalization, the least restrictive measures should be used and regularly reassessed [215,217,303,320,321].

### 5.4. Future Directions and Research Priorities

Despite growing interest in perioperative brain health, patients with dementia remain substantially underrepresented in perioperative research. Many clinical studies exclude individuals with established cognitive impairment, frequently using a Mini-Mental State Examination score below 24 as an exclusion criterion. Consequently, current evidence and clinical guidelines are largely derived from healthier older populations and may not be directly applicable to patients with dementia, who represent those at greatest perioperative risk [322].

Future studies should specifically include patients across the full spectrum of dementia severity and use standardized methods to characterize cognitive status before surgery. Better stratification according to dementia subtype, disease severity, frailty, and functional status would improve the interpretation of postoperative outcomes and facilitate individualized perioperative risk assessment [323,324].

Current evidence is further limited by the predominance of retrospective studies and procedure-specific cohorts, particularly in hip fracture surgery, with relatively little information available for other major surgical procedures. Many studies also lack standardized preoperative cognitive assessments and focus primarily on mortality or short-term complications rather than outcomes that are highly relevant to patients with dementia, including preservation of cognition, functional independence, quality of life, caregiver burden, and the ability to return to previous living arrangements [215,323,324].

The most important unresolved question is whether surgery or anaesthesia produces a sustained change in the natural cognitive and functional trajectory of patients with established dementia. Addressing this question requires prospective longitudinal studies that enroll patients before surgery; characterize baseline dementia severity, subtype, cognitive function, frailty, functional status, and comorbidity burden; and perform repeated assessments after surgery over clinically meaningful follow-up periods. Appropriately matched non-surgical comparison groups are essential for distinguishing postoperative changes from the expected progression of dementia. Studies should also account for delirium, mortality, institutionalization, and loss to follow-up, which may substantially influence observed trajectories. Biomarkers, neuroimaging, and EEG may provide mechanistic information and improve risk stratification, but these approaches should complement rather than replace longitudinal assessment of patient-centred cognitive and functional outcomes [324].

## 6. Conclusions

Patients with dementia represent one of the most vulnerable populations undergoing surgery because of reduced cognitive reserve, ongoing neurodegeneration, chronic neuroinflammation, cerebrovascular dysfunction, and impaired physiological resilience. These factors increase susceptibility to perioperative neurocognitive disorders, particularly postoperative delirium, and contribute to poorer long-term cognitive, functional, and survival outcomes than those observed in cognitively healthy older adults.

Patients with dementia are at increased risk of postoperative delirium, delayed cognitive and functional recovery, institutionalization, and mortality. Current evidence indicates that postoperative delirium may represent an important intermediary pathway linking perioperative stressors with subsequent cognitive and functional deterioration. However, although biological mechanisms involving systemic inflammation, blood–brain barrier dysfunction, mitochondrial injury, neurotransmitter imbalance, and impaired synaptic plasticity are well described, a direct causal relationship between anaesthesia or surgery and accelerated progression of dementia remains unproven. Most available evidence is observational and heterogeneous, and frequently excludes patients with established dementia, limiting the strength and generalizability of current conclusions.

Long-term outcomes are influenced not only by perioperative management but also by baseline dementia severity, frailty, comorbidity burden, functional status, and postoperative complications. Consequently, perioperative care should extend beyond successful completion of surgery to include preservation of cognitive function, functional independence, quality of life, and patient-centred goals of care. Comprehensive multidisciplinary management, including preoperative risk assessment, individualized intraoperative care, prevention and early treatment of postoperative delirium, optimization of physiological homeostasis, rehabilitation, and active involvement of family caregivers, offers the greatest opportunity to improve outcomes in this high-risk population.

Future progress will depend on well-designed prospective studies that include patients across the spectrum of dementia severity and incorporate standardized cognitive and functional assessments, validated biomarkers, neuroimaging, and long-term follow-up. Such studies are essential to clarify the effects of anaesthesia and surgery on dementia trajectories and to support the development of evidence-based, individualized perioperative strategies that preserve both surgical safety and brain health in an increasingly ageing population.

## Figures and Tables

**Table 1:** Perioperative physiological disturbances and their potential relevance in patients with dementia.

Perioperative Factor	Proposed Mechanism	Evidence Specifically in Patients with Established Dementia	Evidence for Delirium/Short-Term Cognitive Outcomes	Evidence for Long-Term Cognitive or Functional Outcomes	Potential Clinical Implications
Hypotension	Reduced cerebral perfusion due to impaired cerebrovascular autoregulation; reduced oxygen and glucose delivery; mitochondrial dysfunction; oxidative stress; neuroinflammation; BBB disruption; synaptic dysfunction [101,102,103,104,105,106,107,108,109,110,111,112,113,114,115,116]	Patients with dementia may have reduced cerebrovascular reserve due to small-vessel disease, endothelial dysfunction, cerebral amyloid angiopathy, and impaired neurovascular coupling, potentially increasing vulnerability to hypotension [106,107]. However, direct clinical evidence linking intraoperative hypotension to accelerated dementia progression is limited [117,118,119].	Cerebral hypoperfusion may contribute to postoperative neurocognitive disorders and impaired postoperative recovery [112,113,114,115,116].	Evidence that brief or transient intraoperative hypotension causes sustained cognitive decline or accelerates underlying neurodegenerative disease in patients with dementia remains limited [117,118,119].	Avoid prolonged or repeated hypotension; use individualized haemodynamic management and maintain adequate cerebral perfusion. Optimal BP targets specifically for patients with dementia remain uncertain [120,121,122,123,124,125,126].
Hypoxia/hypoxaemia	Reduced oxygen availability, impaired aerobic metabolism, ATP depletion, oxidative stress, neuroinflammation, synaptic dysfunction, and neuronal injury; potential effects on amyloid-β and tau pathology [127,128,129,130,131,132,133,134,135,136]	Patients with dementia may have increased vulnerability because of impaired cerebrovascular function, reduced mitochondrial efficiency, and increased oxidative stress [94,95,96,97]. Most evidence regarding neurodegenerative mechanisms is experimental [132,133,134,135,136].	Hypoxaemia may contribute to acute neuronal dysfunction and postoperative neurocognitive vulnerability, although direct evidence in established dementia is limited [130,131,132,133,134].	Direct clinical evidence that brief perioperative hypoxaemia accelerates dementia progression is limited; experimental findings cannot be directly extrapolated to long-term human outcomes [132,133,134,135,136].	Continuous oxygenation monitoring, prompt recognition and correction of hypoxaemia, and careful perioperative respiratory management are appropriate components of brain-protective care [137,138,139].
Carbon dioxide disturbances	Hypocapnia causes cerebral vasoconstriction and may reduce cerebral blood flow; hypercapnia causes vasodilation and may increase cerebral blood volume. Abnormal CO_2_ may also affect neuronal activity, metabolism, and inflammatory responses [140,141,142,143,144,145,146,147,148,149].	Cerebrovascular dysfunction, cerebral amyloid angiopathy, and impaired neurovascular coupling in dementia may increase sensitivity to changes in PaCO_2_ [105,106]. However, direct evidence in established dementia is limited.	Significant disturbances in CO_2_ may alter cerebral perfusion and neuronal function, potentially contributing to perioperative cognitive vulnerability; direct evidence for delirium in dementia is limited [143,144,145,146,147,148,149,150,151].	There is insufficient clinical evidence that perioperative CO_2_ fluctuations cause persistent cognitive decline or accelerate dementia progression [148,149,150,151].	Avoid unnecessary fluctuations in PaCO_2_ and maintain near-normocapnia, particularly in patients with impaired cerebrovascular reserve [149,150,151].
Metabolic disturbances	Hypoglycaemia causes inadequate neuronal glucose supply; hyperglycaemia promotes oxidative stress, inflammation, and potentially worsened ischaemic injury. Mitochondrial dysfunction may increase neuronal susceptibility to injury [152,153,154,155,156,157,158,159,160,161].	Patients with dementia may have impaired cerebral glucose utilisation and mitochondrial dysfunction, potentially increasing vulnerability to metabolic disturbances [157,158]. Evidence directly involving established dementia remains limited.	Major glucose disturbances may impair cognitive function and neurological recovery in older patients, but dementia-specific evidence is limited [154,155,156].	Experimental evidence supports potential neuronal injury mechanisms, but there is insufficient evidence that perioperative metabolic disturbances accelerate long-term dementia progression in humans [159,160,161].	Avoid major hypo- and hyperglycaemia and maintain metabolic homeostasis, particularly in older adults with dementia and multimorbidity [162,163,164,165,166,167,168,169].
Temperature dysregulation	Hypothermia alters coagulation, drug metabolism, immune function, sympathetic activity, and tissue oxygen delivery; experimental data suggest effects on neuroinflammation, oxidative stress, and neuronal metabolism [170,171,172,173,174,175]. Hyperthermia increases cerebral metabolic demand and may amplify inflammatory responses and neuronal injury [176,177].	Older adults with dementia may have increased vulnerability because of impaired thermoregulation, autonomic dysfunction, frailty, and reduced physiological reserve [169,170]. Direct dementia-specific evidence remains limited.	Perioperative temperature disturbances may adversely affect physiological recovery; fever and infection may contribute to postoperative neurological dysfunction [165,166,167,168,169,170,171,172,173,174,175,176,177].	Evidence directly linking mild perioperative hypothermia or temperature management to persistent cognitive decline or dementia progression is limited [174,175,176,177,178,179,180].	Continuous temperature monitoring and active maintenance of normothermia are recommended. Both hypothermia and hyperthermia should be avoided as part of general brain-protective perioperative care [178,179,180,181,182].

## Data Availability

No new data were created or analyzed in this study. Data sharing is not applicable to this article.

## Abbreviations

The following abbreviations are used in this manuscript:

ACP	Advance Care Planning
ADLs	Activities of Daily Living
ADRD	Alzheimer’s Disease and Related Dementias
BPSD	Behavioral and Psychological Symptoms of Dementia
BSR	Burst Suppression Ratio
DoA	Depth of Anesthesia
DSD	Delirium Superimposed on Dementia
EEG	Electroencephalography
ESAIC	European Society of Anaesthesiology and Intensive Care
GABA	Gamma-Aminobutyric Acid
HMGB1	High-Mobility Group Box 1
IADLs	Instrumental Activities of Daily Living
IL-1β	Interleukin-1 Beta
IL-6	Interleukin-6
MDT	Multidisciplinary Team
MMSE	Mini-Mental State Examination
MTBC	Multidisciplinary Team-Based Care
PND	Perioperative Neurocognitive Disorders
POCD	Postoperative Cognitive Dysfunction
POD	Postoperative Delirium
POQI	PeriOperative Quality Initiative
PP2A	Protein Phosphatase 2A
ROS	Reactive Oxygen Species
TNF-α	Tumor Necrosis Factor Alpha
